# Case Report: A potential novel treatment for drug-induced acute interstitial nephritis: Benralizumab

**DOI:** 10.3389/fimmu.2025.1720992

**Published:** 2025-11-17

**Authors:** Alexander Saadia, Ron Wald, Assaf Potruch, Galina Pizov, Karen Meir, Kobi Gorin, Inon Sarig, Eyal Ben Dori, Limor Rubin, Yaarit Ribak, Odded Shamriz, Yuval Tal, Aviv Talmon

**Affiliations:** 1 Department of Nephrology and Hypertension, Hadassah Medical Center, Faculty of Medicine, Hebrew University of Jerusalem, Jerusalem, Israel; 2 Division of Nephrology, St. Michaels Hospital, Toronto, ON, Canada; 3 Department of Pathology, Hadassah Medical Center, Faculty of Medicine, Hebrew University of Jerusalem, Jerusalem, Israel; 4 Department of Endocrinology and Metabolism, Hadassah Medical Center, Faculty of Medicine, Hebrew University of Jerusalem, Jerusalem, Israel; 5 Allergy and Clinical Immunology Unit, Hadassah Medical Center, Faculty of Medicine, Hebrew University of Jerusalem, Jerusalem, Israel

**Keywords:** acute interstitial nephritis (AIN), IL-5 antibody, benralizumab, dialysis, acute kidney injury

## Abstract

A 55-year-old man presented to the emergency department (ED) with diffuse abdominal pain, vomiting, and anuria following a night of heavy alcohol intake. The patient’s medical history included hypertension. Two weeks prior to presentation, he was prescribed etoricoxib for back pain. On presentation, he was found to have severe acute kidney injury, and dialysis was initiated. Renal biopsy showed acute interstitial nephritis with numerous eosinophilic infiltrates. Despite stopping etoricoxib and initiating high-dose steroids, kidney function did not improve. The administration of benralizumab—an anti-CD125 antibody—resulted in rapid and complete recovery of kidney function. This case report highlights the potential role of benralizumab in the treatment of drug-induced interstitial nephritis with eosinophiluria.

## Case presentation

A 55-year-old male presented to the emergency department (ED) after three days of diffuse abdominal pain, diarrhea, vomiting, diminished oral intake, and anuria. The symptoms followed a night of substantial alcohol consumption. His medical history included hypertension treated with ramipril and amlodipine, metabolic dysfunction-associated steatotic liver disease, and impaired glucose tolerance. He reported no chronic use of other nephrotoxic or other prescription medications, over-the-counter drugs, or herbal supplements. Two weeks prior to presentation, he was prescribed etoricoxib 120 mg once daily for lower back pain, and laboratory tests performed at the time showed a serum creatinine of 0.75 mg/dL, blood urea nitrogen (BUN) 14 mg/dL, sodium 139 mmol/L, and potassium 4.2 mmol/L, indicating normal baseline renal function and electrolyte balance.

Upon arrival at the ED, he was alert and lucid; appearing pale with mild scleral icterus. His temperature was 36.5 °C, blood pressure was 167/92 mmHg, heart rate was 100 beats per minute, respiratory rate was 16 breaths per minute, and O_2_ saturation was 96% on room air. Pulmonary, cardiovascular, and abdominal examinations were unremarkable. No rashes were evident.

Laboratory testing revealed a creatinine of 12.6 mg/dL, serum sodium 125 mmol/L, and elevated cholestatic and hepatic liver enzymes (**Table 1**). Microscopic review of the urine sediment revealed isomorphic red blood cells, numerous white blood cells, and white blood cell casts.

**Table 1:** Laboratory findings of the patient on admission and 48 hours after hospitalization.

Variable	Reference range, Adult males*	Upon presentation to the emergency department	48 hours following presentation
Hemoglobin (g/dL)	13.9 - 17.7	13.9	12
Hematocrit (%)	39.6 - 51.8	40.3	34.5
White-cell count (per µl)	3.79 - 10.33	8.1	6.7
Differential count (absolute per µl)			
Neutrophils	1.78 - 7	6.3	4.4
Lymphocytes	1.07 - 3.12	0.9	1.1
Monocytes	0.24 - 0.73	0.6	0.7
Eosinophils	0.03 - 0.47	0.0	0.2
Platelet count (per µl)	166 - 389	155	140
Sodium (mmol/L)	136 - 145	125	130
Potassium (mmol/L)	3.5 - 5.1	4.6	4.3
Chloride (mmol/L)	98 - 107	92	95
Blood urea nitrogen (mg/dL)	9 - 23	74.6	78.8
Creatinine (mg/dL)	0.7 - 1.3	12.6	16.45
Glucose (mg/dL)	74 - 106	89	114
Lacatate dehydrogenase (IU/L)	110 - 210	273	203
Calcium (mg/dL)	8.3 - 10.6	8.3	7.2
Phosphorus (mg/dL)	2.4 - 5.1	5.4	9.5
Alanine aminotransferase (IU/L)	10 - 49	2571	736
Aspartate aminotransferase (IU/liter)	0 - 34	203	17
Total bilirubin (mg/dL)	0.3 - 1.2	2.06	0.54
Conjugated bilirubin (mg/dL)	0 - 0.3	1.31	0.36
Diastase (u/L)	30-118	43	
Albumin (g/dL)	3.2 - 4.8	4.1	3.6
High-sensitivity troponin T (ng/L)	0 - 53		12.96
Lactate (mg/dL)	0 – 0.5	23.6	6.1
C-reactive protein (mg/dL)	0 – 0.5	1.33	1.18
Arterial blood gases			
Fraction of inspired oxygen		21%	21%
pH	7.35 - 7.45	7.288	7.355
Partial pressure of carbon dioxide (mmHg)	30 - 40	39.5	17.4
Partial pressure of oxygen (mmHg)	30-40	22.8	32.9
Bicarbonate (mmol/L)	18 - 24	18.5	17.4
Base excess (mmol)	-2 - 2	-7.6	-6.9

*Reference values are affected by many variables, including the patient population and the laboratory methods used. The ranges used at our institution, Hadassah Medical Center, Jerusalem are for male adults who do not have medical conditions that could affect the results. They may therefore not be appropriate for all patients.

The urine toxicology screen was negative for illicit or over-the-counter drugs.

## Course in hospital

The patient was admitted to the Internal Medicine service. Given his exposure to etoricoxib and urinalysis findings, acute interstitial nephritis (AIN) was suspected as the leading cause of acute kidney injury (AKI). The patient was clinically volume contracted on presentation, and 0.9% sodium chloride was administered. However, despite receiving 5 L of fluid over two days and showing evidence of volume repletion, he remained anuric. Renal replacement therapy (RRT) was initiated three days after hospitalization.

Serology for hepatitis A, B, and C, as well as HIV, were negative for acute or chronic infection as was an autoimmune panel that included antibodies for ANA and anti-smooth muscle. Tests for anti-parietal cells, anti-mitochondria, anti-proteinase 3, and anti-myeloperoxidase were negative. C3 and C4 were normal. Immunoglobulin A, G, and M levels were within the normal range. Serum protein electrophoresis was negative for monoclonal gammopathy, and the free kappa:lambda ratio was 0.71 (normal range 1.56–0.31).

Given the recent alcohol intake and the patient’s known steatotic liver disease, acute hepatocellular or cholestatic injury was initially considered in the differential diagnosis, along with possible pancreatic involvement. The findings were consistent with acute alcoholic hepatitis and a component of dehydration, as imaging revealed normal hepatic, pancreatic, and biliary morphology, and liver enzyme levels declined rapidly within 24 hours of fluid resuscitation. However, despite this rapid hepatic improvement, serum creatinine remained markedly elevated and the patient remained anuric, suggesting that the acute kidney injury was unlikely to be solely secondary to liver dysfunction or alcohol intoxication.

Unenhanced computed tomography (CT) of the chest, abdomen, and pelvis revealed no evidence of nephrolithiasis or kidney obstruction. The kidneys measured 10.5 cm and 11 cm on the right and left sides, respectively, without thinning of the renal cortex. The liver had a normal appearance. Chest CT showed small bilateral pleural effusions without other remarkable findings.

**Figure 1:**
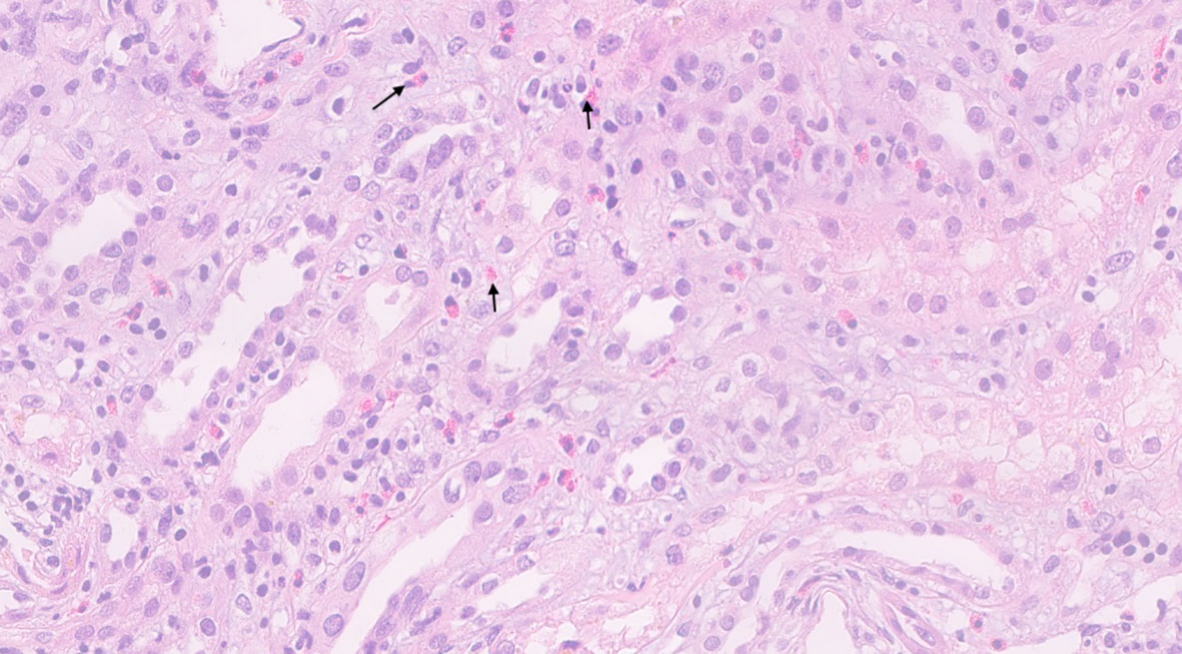
Hematoxylin & eosin stain of the kidney biopsy. Inflammatory cells are present in the interstitium of the kidney with prominent eosinophils (arrows) seen distributed across the kidney parenchyma.

A renal biopsy was performed. Of the 22 cortical glomeruli, one was sclerotic. Light microscopy with hematoxylin and eosin staining showed an interstitial inflammatory infiltrate consisting of mononuclear cells with numerous eosinophils infiltrating approximately 30% of the cortex. There were signs of acute tubular injury and focal tubular accumulation of necrotic proteinaceous material and Tamm–Horsfall protein. Glomeruli were normal without mesangial proliferation, inflammatory infiltrates, or focal damage. Immunofluorescence results were negative for IgG, IgA, IgM, C3, and C1q. The final diagnosis was acute interstitial nephritis (**Figure 1**).

The patient was started on oral prednisone 80 mg (1 mg/kg) daily for four days, followed by intravenous methylprednisolone 250 mg daily for two days. This escalation was chosen due to the lack of clinical response to oral corticosteroids and concern about the severity of kidney injury. However, urine output remained below 100 mL per day, and the patient remained dialysis-dependent.

**Figure 2:**
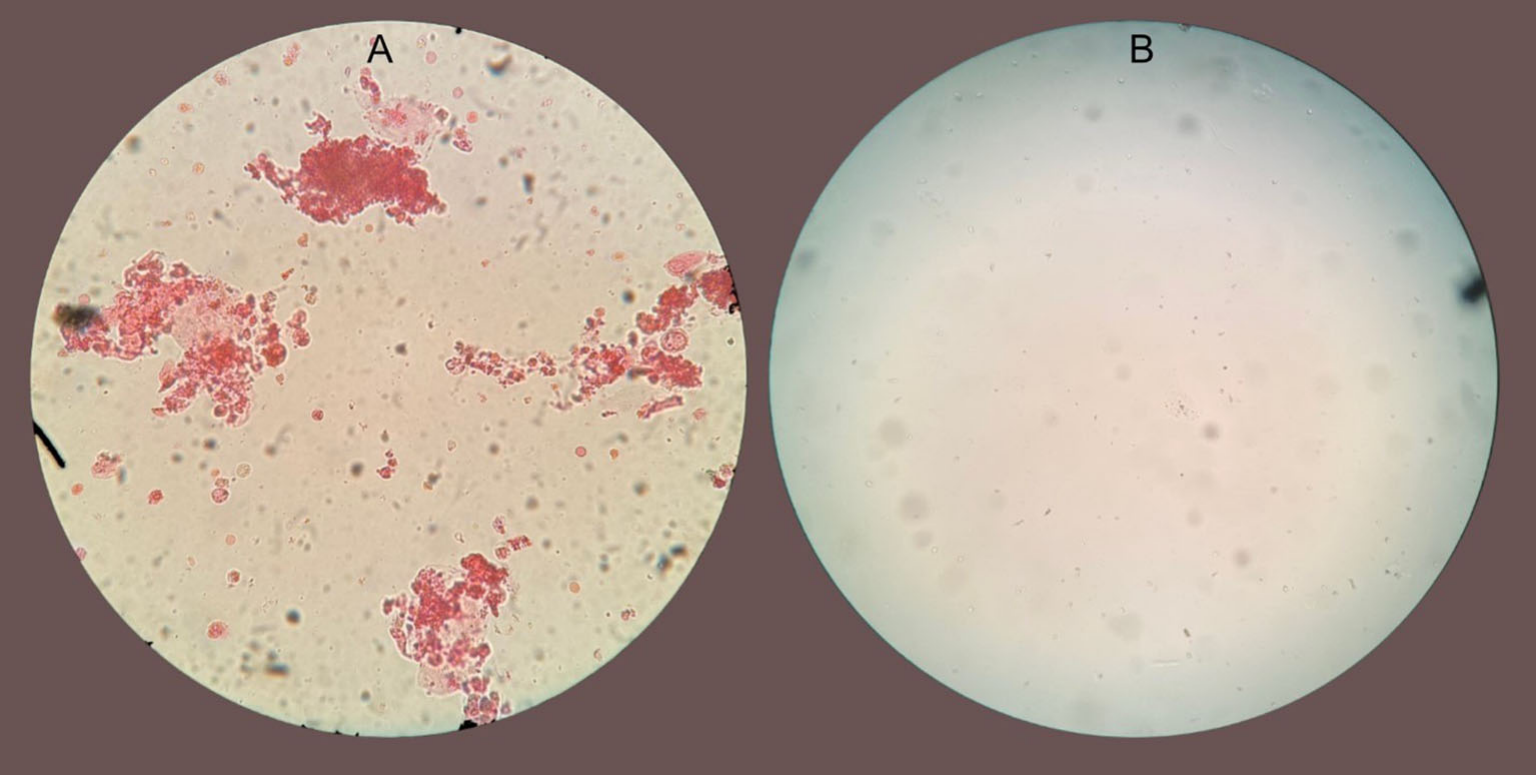
**(A)** Microscopy of urinary sediment at 40x magnification stained with eosin showing clumps of mostly eosinophilic cells with eosinophil clumping. **(B)** Microscopy of urinary sediment stained with eosin from the same patient 48 hr after benralizumab.

A repeat urinalysis revealed persistent leukocyturia and white blood cell casts. Considering that the biopsy showed significant eosinophil infiltration, we suspected that eosinophils would also be present in the urine sediment. Eosin staining of urine confirmed that almost all urinary leukocytes and casts were eosinophils (**Figure 2a**). Because Hansel’s stain was not available at our institution, eosinophil identification was performed using a 1% eosin stain obtained from the pathology laboratory, which, although less sensitive than Hansel’s method, allowed satisfactory visualization of eosinophilic granules. The stained sediment was photographed and reviewed by a renal pathologist, who confirmed the morphology of eosinophils.

The continued presence of eosinophiluria despite high-dose steroids suggested a pathogenic role for eosinophils. This led us to consider benralizumab, a monoclonal antibody targeting the alpha chain of the interleukin-5 receptor (CD125) on eosinophils. After obtaining informed consent for off-label use by our institution’s Internal Review Board we administered a single 30 mg subcutaneous dose of benralizumab on day 10 of hospitalization.

**Figure 3:**
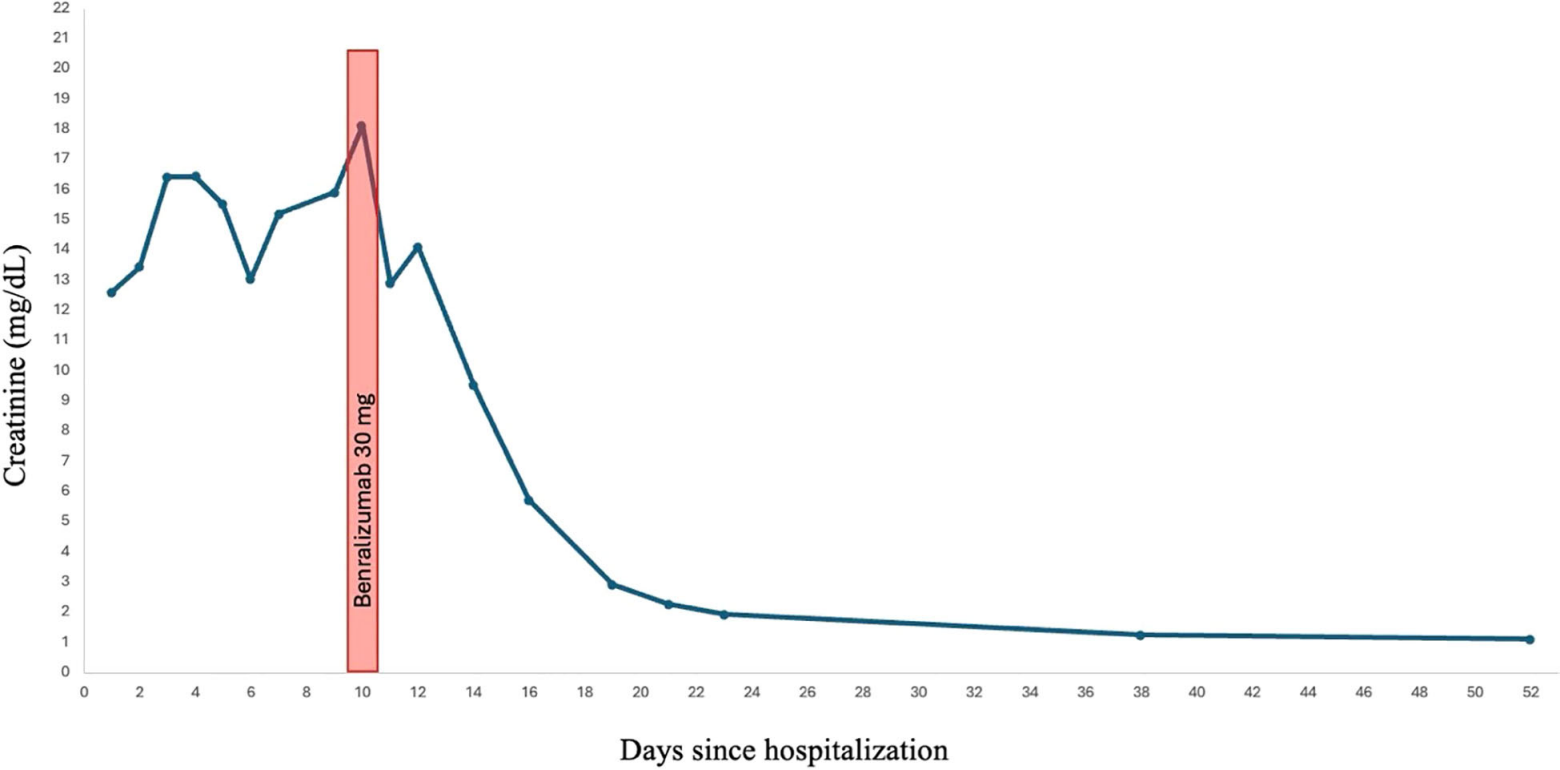
Creatinine decrease following benralizumab treatment.

Within 12 h of receiving benralizumab, the patient was no longer anuric and had produced approximately 1.5 liters of urine. In the ensuing days, urine output increased to 3–4 liters per day. A week after receiving benralizumab, a creatinine clearance of 75 mL/min was measured from a 24-hour urine collection (**Figure 3**). He was weaned off dialysis after three more sessions, and prednisone was tapered and discontinued completely after six weeks of treatment. Further administration of benralizumab was deemed unnecessary.

Kidney function returned to his previous baseline after one month, and repeat urinary dipsticks were negative for leukocyturia, hematuria, and proteinuria during follow-up after discharge.

## Discussion

We describe a case of severe etoricoxib-induced acute interstitial nephritis with eosinophiluria in which kidney function improved rapidly following the administration of benralizumab.

AIN is present in up to 19% of all biopsies for AKI. In 70–75% of cases, AIN is drug-induced, with NSAIDs being a frequent culprit (1). The diagnosis is often presumptive, as discontinuation of the suspected drug is both diagnostic and therapeutic, leading to low rates of kidney biopsies in patients with AIN. Oliguria or anuria may occur in up to 51% of AIN cases, and 10–15% of patients will require RRT, and recovery of renal function typically takes weeks to months (2). AIN can result in interstitial fibrosis and chronic kidney damage (3). In the absence of clinical recovery after discontinuing the culprit drug, glucocorticoids are the mainstay of therapy, although the triggers for initiation, optimal dose, duration of therapy, and even their effectiveness remain controversial (4–6). Other than glucocorticoids and supportive kidney care, no other treatment options for AIN have proven to be effective.

Benralizumab was approved by the U.S. Food and Drug Administration for the treatment of eosinophilic asthma (7, 8). It is also used empirically for patients in whom the pathophysiologic mechanism of the disease is thought to be eosinophil-mediated (9). In most cases, benralizumab is administered to patients with substantial peripheral eosinophilia, but it has also been used successfully in cases without eosinophilia (10). Benralizumab blocks the IL-5 receptor on eosinophils, which has two distinct effects on the target cells. First, by blocking IL-5 stimulation, cells cannot function or survive. Second, the antibody opsonizes the receptor, leading to antibody-dependent cell cytotoxicity (ADCC) mediated by natural killer (NK) cells (11). In the context of acute interstitial nephritis, this mechanism could lead to rapid depletion of eosinophils within the renal interstitium, thereby attenuating local cytokine release, reducing tissue edema, and halting ongoing tubulointerstitial injury. The prompt diuresis and recovery observed after benralizumab administration are consistent with this biologically plausible eosinophil-targeted effect.

The patient described in this case did not have peripheral eosinophilia, but the prominence of eosinophils in the kidney interstitium seen in the biopsy, as well as the eosinophiluria observed while the patient was on high-dose steroids, prompted us to try a specific anti-eosinophil medication in an attempt to reduce kidney inflammation and restore kidney function.

Many articles have discussed the lack of diagnostic value of eosinophiluria for diagnosing AIN; however, the actual role of eosinophils has not been fully elucidated (12).

The rapid recovery after benralizumab administration strongly suggests the pathogenic role of eosinophils in drug-induced AIN. As the patient received systemic glucocorticoids before and after benralizumab, it is difficult to definitively attribute kidney recovery solely to benralizumab. It is possible that with a longer observation period after withdrawing the offending drug and continuing glucocorticoid monotherapy, kidney recovery would have occurred without benralizumab. However, the temporal relationship between benralizumab administration and kidney function recovery, along with the disappearance of eosinophils in the urine within 48 hours after the drug was administered (**Figure 2B**), suggests that benralizumab contributed substantially to the resolution of the patient’s AIN, possibly in synergy with corticosteroids. Typically, glucocorticoid-induced improvement in drug-related AIN occurs over several days to weeks rather than within hours, supporting a specific and accelerated effect related to eosinophil depletion by benralizumab. Previous studies have demonstrated that administration of a single dose of benralizumab results in rapid and profound eosinophil depletion, typically within 24 hours (14). This effect remained stable thereafter, likely because the offending agent had been discontinued, and additional doses of benralizumab were not required.

To our knowledge, this is the first reported case of benralizumab being used to treat AIN. This agent should be considered in drug-induced AIN characterized by a hypersensitivity reaction in which eosinophils are likely crucial mediators of injury. Further investigation is needed to establish the role of benralizumab in drug-induced AIN and to determine whether it is a more effective alternative to glucocorticoids. Additionally, further studies could assess eosinophil involvement and activation by measuring tissue or urinary markers such as IL-5 and specific eosinophilic granule contents such as EDN, ECP, EPX, MBP, and others (13).

## Data Availability

The original contributions presented in the study are included in the article/supplementary material. Further inquiries can be directed to the corresponding author.
